# Advance care planning in dementia: recommendations for healthcare professionals

**DOI:** 10.1186/s12904-018-0332-2

**Published:** 2018-06-21

**Authors:** Ruth Piers, Gwenda Albers, Joni Gilissen, Jan De Lepeleire, Jan Steyaert, Wouter Van Mechelen, Els Steeman, Let Dillen, Paul Vanden Berghe, Lieve Van den Block

**Affiliations:** 1 Department of Geriatric Medicine, Ghent University Hospital, Ghent, Belgium; 2 End-of-life Care Research Group, Vrije Universiteit Brussel (VUB) and Ghent University, Laarbeeklaan 103, 1090 Brussels, Belgium; 3 Flanders Federation for Palliative Care, Vilvoorde, Belgium; 4 Department of Public Health and Primary Care, ACHG, KU Leuven, Leuven, Belgium; 5 Department of Sociology, University of Antwerp, Antwerp, Belgium; 6 Flemish Expertise Centre on Dementia Care, Antwerp, Belgium; 7 Academic Centre for Nursing and Midwifery, KULeuven, Leuven, Belgium; 8 Department of Geriatric Medicine, Ghent University Hospital, Ghent, Belgium; 9 Department of Family Medicine and Chronic Care, Vrije Universiteit Brussel (VUB), Laarbeeklaan 103, 1090 Brussels, Belgium

**Keywords:** Advance care planning, Alzheimer’s disease, Dementia, Elderly care, Guideline, Recommendations

## Abstract

**Background:**

Advance care planning (ACP) is a continuous, dynamic process of reflection and dialogue between an individual, those close to them and their healthcare professionals, concerning the individual’s preferences and values concerning future treatment and care, including end-of-life care. Despite universal recognition of the importance of ACP for people with dementia, who gradually lose their ability to make informed decisions themselves, ACP still only happens infrequently, and evidence-based recommendations on when and how to perform this complex process are lacking. We aimed to develop evidence-based clinical recommendations to guide professionals across settings in the practical application of ACP in dementia care.

**Methods:**

Following the Belgian Centre for Evidence-Based Medicine’s procedures, we 1) performed an extensive literature search to identify international guidelines, articles reporting heterogeneous study designs and grey literature, 2) developed recommendations based on the available evidence and expert opinion of the author group, and 3) performed a validation process using written feedback from experts, a survey for end users (healthcare professionals across settings), and two peer-review groups (with geriatricians and general practitioners).

**Results:**

Based on 67 publications and validation from ten experts, 51 end users and two peer-review groups (24 participants) we developed 32 recommendations covering eight domains: initiation of ACP, evaluation of mental capacity, holding ACP conversations, the role and importance of those close to the person with dementia, ACP with people who find it difficult or impossible to communicate verbally, documentation of wishes and preferences, including information transfer, end-of-life decision-making, and preconditions for optimal implementation of ACP. Almost all recommendations received a grading representing low to very low-quality evidence.

**Conclusion:**

No high-quality guidelines are available for ACP in dementia care. By combining evidence with expert and user opinions, we have defined a unique set of recommendations for ACP in people living with dementia. These recommendations form a valuable tool for educating healthcare professionals on how to perform ACP across settings.

## Background

Due to the aging population, the number of people with dementia is increasing. In 2015, the World Health Organization (WHO) estimated the number of people living with dementia at 35.6 million. This is expected to double by 2030 and even triple by 2050 [1].

To enable caregivers to improve the quality of life of people with dementia, they need to know what is important to them, what specific concerns they are facing and how and where they want to receive care. However, people living with dementia gradually lose their ability to make informed decisions themselves [2]. Therefore it may be necessary to have these discussions in the earlier stages of dementia, when the person is still able to make decisions and express their values and preferences [3].

Providing high-quality care for people with dementia requires advance care planning (ACP) [4]. ACP is a continuous, dynamic process of early reflection and dialogue between a person with dementia, those close to them and the relevant healthcare professionals concerning the person’s preferences and values when it comes to future treatment and care, including end-of-life care [5]. If they wish, the contents of these conversations can be recorded in the form of an advance directive, and a proxy decision-maker can be appointed or a permanent power of attorney can be granted in anticipation of future deterioration [6, 7].

Despite the widespread recognition of the importance of ACP for people living with dementia [1–3, 8–10], the reality is different, and only a minority of people with dementia get the opportunity to engage in ACP [11]. For example, studies show that a minority of deceased nursing home residents with dementia had an advance directive [12–14] and that general practitioners (GPs) had communicated infrequently about future end-of-life care options. For example, only 22% of deceased nursing home residents in Belgium had an ACP conversation [13]. Even among a representative sample of non-sudden deaths in Belgium and the Netherlands, only 34% of patients had engaged in ACP with their GP [15]. People with dementia are often a disadvantaged group when it comes to being invited for ACP conversations at an appropriate time and cognitive decline is often seen as a barrier to initiate ACP [16–26].

Although several organisations and professionals have called for guidance on when and how to perform ACP in this specific population [1–3], guidelines that have been developed in a systematic way using the best evidence available are lacking. In an attempt to improve the prevalence, quality and consistency of ACP in people with dementia, we aimed to develop clinical recommendations for applying and conducting ACP in practice, to provide support for healthcare staff (physicians, including GPs, nurses, allied health and care workers) who work with people living with dementia in the community, residential and hospital settings.

## Methods

No informed consent was needed for this study. The procedure developed by the Belgian Centre for Evidence-Based Medicine (CEBAM) (in close cooperation with the Belgian Federal Public Service Health, Food Chain Safety and Environment and the two professional Belgian GP organisations Domus Medica and Société Scientifique de Médecin General or SSMG) was used as methodology to develop a guideline. The procedure entails: 1) a literature search to identify what is already known about ACP in people living with dementia, 2) the development of recommendations based on the existing evidence and expert opinion of the author group, and 3) a validation process to provide feedback on the clarity, acceptability and importance and to discuss possible barriers to implement the recommendations.

### 1) Literature search

#### Search framework: selection of research questions and clinical themes on ACP and clinical practice

A multidisciplinary group of authors was assembled to develop the recommendations: a research coordinator, a geriatrician, two GPs, an expert in dementia care, a nurse, two psychologists and the director of the Flanders Federation for Palliative Care. Collectively, they have extensive experience in palliative, primary and dementia care in different settings in Flanders (Belgium). Based on their own experience and obstacles they have encountered in practice, this author group formulated clinical research questions related to ACP in people with dementia. Obstacles are defined as those areas in the ACP process that cause ACP not to be initiated at the appropriate time or not to be performed at all. The clarity, applicability and completeness of each clinical research question was evaluated in semi-structured interviews by SM and DN with 28 GPs, of whom 14 also act as coordinating advisory physicians or CAPs in a nursing home and of whom 14 are heads of residential care, all from different nursing homes in Flanders and all familiar with the concept and practice of ACP. Through discussion and consensus within the team of authors, these were categorized to six main clinical themes to guide the literature search (see Table 1).

**Table 1:** The six clinical themes and examples of research questions used to search for evidence

Theme 1	Mental capacity
E.g.:	How can mental capacity be defined in the context of healthcare for people living with dementia?
	How can mental capacity be evaluated?
Theme 2	Advance care planning in people living with dementia
E.g.:	What are the specific points of interest in the involvement of people living with dementia in advance care planning? For early stages: How do we deal with persons who lack disease insight? What if people are resistant to talk about future care? For mild stages of dementia in whom verbal communication is still possible? For people with dementia in whom verbal communication about ACP is too difficult or not possible
	What if the wishes of the mentally competent person (the ‘then self’) does not correspond to the actual wishes of the person now lacking in mental capacity (the ‘now self’) or to the ‘best interests’ of the person?
Theme 3	Family and environment of people living with dementia
E.g.:	What is the role of family and the immediate social circle in advance care planning throughout the different stages of dementia?
	How can healthcare professionals support families and those in the person’s immediate environment in taking on these roles?
Theme 4	Specifics for advance care planning in people living with early onset dementia
E.g.:	Are there specific points of interest concerning people living with early onset dementia and advance care planning?
Theme 5	Documentation and registration of ACP
E.g.:	What aspects of ACP need to be registered? How do we transfer information to different settings?
Theme 6	Organizational issues
E.g.:	What is the role in the ACP process of different professionals? What are the optimal preconditions for ACP in different settings?

#### Search for evidence

We undertook a stepwise approach to search the scientific literature for evidence about ACP in people with dementia related to the six selected clinical themes. Publications were included if (i) they were published in Dutch, French, English or German, (ii) their main theme was ACP in people with dementia, or, if a guideline, ACP was included in the goals, or was one of the outcome recommendations. Publications were excluded if ACP in people with dementia was not the focus of the article. Meta-analyses and systematic reviews were excluded if they were published before 2004, to avoid including publications that approach ACP too narrowly. The authors believe that the majority of publications only started defining ACP from 2004 onward as a more comprehensive process that is not limited to advance directives [27].

**Fig. 1:**
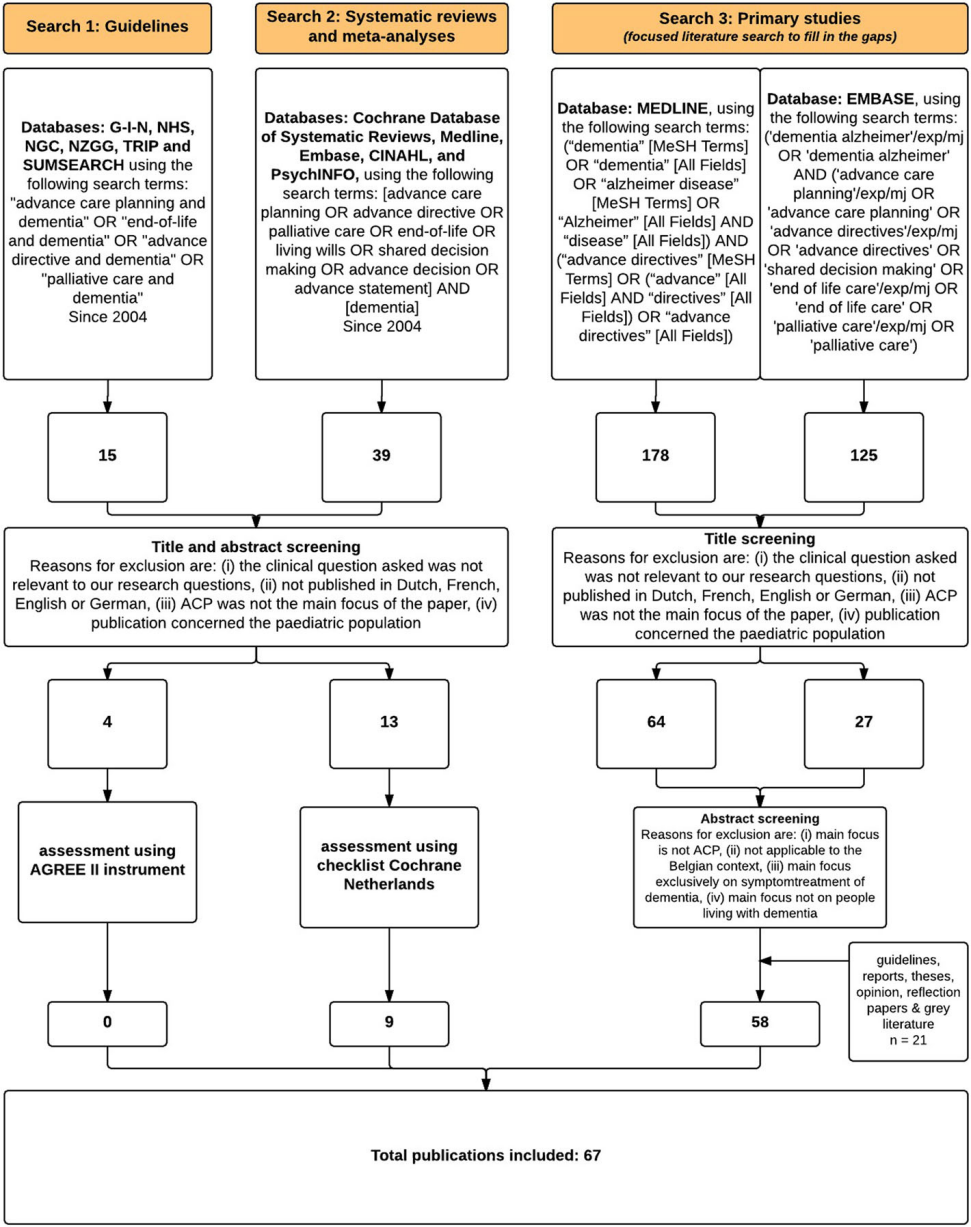
PRISMA flow diagram of the study screening, eligibility, selection and inclusion process ACP advance care planning; G-I-N Guidelines International Network; NHS National Health Service; NGC National Guideline Clearinghouse; NZGG New Zealand Guidelines Group; TRIP Trip medical database; MeSH Medical Subject Headings; AGREE Appraisal of Guidelines for Research and Evaluation

The search consisted of three steps to identify relevant 1) international guidelines, 2) systematic reviews and meta-analysis and 3) primary studies (randomised controlled trials and observational research). Search terms and a PRISMA flowchart are provided in Fig. 1. The quality assessment procedure is described below.

1. We searched for existing guidelines concerning ACP and dementia in guideline databases G-I-N (Guidelines International Network), NHS (National Health Service), NGC (National Guideline Clearinghouse), and a databank of the NZGG (New Zealand Guideline Group), making use of two EBM-search engines (TRIP and SUMSEARCH).
2. Systematic reviews and meta-analyses were searched for by two authors (SM and DN) using five major bibliographic databases (Cochrane Database of Systematic Reviews, Medline, Embase, CINAHL and PsycINFO).
3. Two authors independently performed a *focused* literature search in Medline and Embase for primary studies (randomised controlled trials or observational studies) to answer clinical questions which could not be answered through guidelines, systematic reviews and/or meta-analysis. In addition, by using the snowballing method and based on expert advice, additional articles that may have been missed were added if perceived relevant by the authors.

#### Quality assessment

The quality of the guidelines and systematic reviews and/or meta-analyses was independently checked by pairs of authors against the Appraisal of Guidelines Research and Evaluation (AGREE II) instrument [28] for guidelines and a checklist to assess the methodological quality of systematic reviews [29], as recommended by CEBAM and Cochrane Netherlands [30–35]. The AGREE II instrument is an international 23-item tool to assess the quality and reporting of practice guidelines that is organised into six domains. All items are rated on a 7-point scale (1 ‘strongly disagree’ – 7 ‘strongly agree’). The checklist used for assessment of systematic reviews was developed by Cochrane Netherlands (http://netherlands.cochrane.org; only available in Dutch). It has been shortened by the authors. It now consists of 12 ‘yes – no – cannot answer/too little information in the paper’ questions, organised into three categories: validity, importance and applicability. Questions for assessment include: 1) Was the search request adequately formulated? 2) Was the search performed adequately? 3) Was the selection procedure for the articles performed adequately? 4) Was the quality assessment performed adequately? 5) Is the description of how the data extraction was organised adequate? 6) Were the most important characteristics of the included research reported? 7) Was the meta-analysis carried out appropriately? 8) Is there statistical pooling? 9) Is the research valid? 10) Are the results adequately described? 11) Are the findings applicable in the region? 12) Is this applicable in daily practice? (translation by authors). The quality score is the number of times ‘yes’ was applied to the questions (1 ‘low quality’ - 12 ‘high quality’). As specified by the CEBAM procedure, we would have followed the scientific process of the ADAPTE procedure when the AGREE assessment was performed, to adapt useful guidelines to the local context through a process that can be found elsewhere [31–35]. However, none of the guidelines met these criteria and because of the limited number of systematic reviews on ACP and dementia the authors decided to include other primary studies (randomised controlled trials and observational research), opinion pieces and grey literature as well. An overview of all included publications is provided in Table 2.

**Table 2:** Overview and characteristics of publications included (*n* = 67)

Systematic reviews and meta-analysis (*n* = 9)					
First author (year of publication)		Study type	Number of publications included (n)	Quality score ranging from 1 to 12* (number of items that could not be answered due to too little information in the paper)	
1	Dening (2011)	Review	17	8 (4)	
2	Robinson (2012)	Systematic review	4	7 (5)	
3	Seeber (2012)	Review	43	6 (5)	
4	Van der Steen (2010)	Systematic review	45	4 (8)	
5	Sampson (2010)	Review (editorial)		2 (2)	
6	Goodman (2010)	Integrative review	68	8 (3)	
7	De Boer (2010)	Literature review	*Information not available*	3 (7)	
8	Raymond (2014)	Critical synthesis	8	6 (3)	
9	Van der Steen (2014)	Systematic review	33	7 (4)	
Other (*n* = 58)					
First author (year of publication)		Methods	Setting (sample, n)		
*Quantitative and experimental research*					
1	Detering (2010)	Randomised controlled trial	Medical inpatients aged 80 or more (*n* = 309)		
2	Vandervoort (2012)	Cross-sectional retrospective survey	Deceased residents with dementia in 345 nursing homes (*n* = 764)		
3	De Gendt (2010)	Cross-sectional retrospective survey	Nursing home administrators (*n* = 345)		
4	Benkendorf (1997)	Prospective cohort study	Patients > or = 19 years old with arrest of presumed cardiac cause, with locations at home or at a nursing home (*n* = 2348)		
5	De Gendt (2013)	Cross-sectional retrospective survey	Deceased nursing home residents (*n* = 1240)		
6	Sampson (2011)	Exploratory randomised controlled trial	Family caregivers of patients with severe dementia (*n* = 33; IG: *n* = 22; CG: *n* = 11)		
7	Brazil (2015)	Cross-sectional survey	General practitioners (*n* = 133)		
8	Grisso (1997)	Quasi-experimental trial	Acutely ill inpatients with a diagnosis of schizophrenia or schizoaffective disorder (IG: *n* = 40)		
9	Givens (2009)	Prospective cohort study	Nursing home residents with advanced dementia and their healthcare proxies (*n* = 223)		
10	Vandervoort (2013)	Cross-sectional retrospective survey	Deceased residents with dementia in 69 nursing homes (*n* = 198)		
11	Baile (2002)	Questionnaires	Oncologists (*n* = 167)		
12	Szafara (2012)	Prospective cohort study	Residents (*n* = 1044 US, *n* = 513 Netherlands)		
13	van der Steen (2012)	Prospective cohort study	Residents with advanced dementia (*n* = 94)		
*Qualitative research*					
14	Garand (2011)	Semi-structured interviews	Persons (*n* = 127) with a diagnosis of MCI or early AD (*n* = 72) or moderate to severe AD (*n* = 55)		
15	de Boer (2012)	Semi-structured interviews	Individuals diagnosed with early-stage AD (*n* = 24)		
16	Poppe (2013)	In-depth interviews	Patients with memory problems or mild dementia (n = 2) and eight carers (*n* = 8) and staff members from a memory clinic and a community mental health team (*n* = 11)		
17	Chan (2011)	Semi-structured interviews	Nursing home residents (*n* = 42)		
18	Piers (2013)	Semi-structured interviews	Elderly patients with limited prognosis (*n* = 38)		
19	Ashton (2014)	Interviews	Family caregivers within a specialist dementia unit (*n* = 12)		
20	Levi (2010)	Focus groups	Older individuals (*n* = 23)		
21	Kim Suh (2011)	Interviews	Persons with AD (*n* = 188)		
22	Shanley (2009)	Interviews	Managers from residential aged care facilities (*n* = 41)		
23	Dening (2012)	Nominal group study	People with dementia (n = 6), carers (*n* = 5) and dyads of people with dementia and carers (*n* = 6) attending memory assessment services		
24	Dening (2012)	Whole-systems qualitative study based on interviews and focus groups	Nine carers of people with dementia (*n* = 9) and focus groups (*n* = 6) with health care professionals with mixed professions (*n* = 26) and individual interviews with health care professionals with mixed professions (*n* = 15)		
25	Hirschman (2006)	Semi-structured interviews	Family members of patients with advanced dementia (n = 30)		
26	Hirschman (2008)	Semi-structured interviews	Family members of patients with advanced dementia (*n* = 30)		
27	Dickinson (2013)	Semi-structured interviews	People with mild to moderate dementia (*n* = 17) and family carers (*n* = 29)		
28	Hoe (2007)	Semi-structured interviews	Care recipient and caregiver dyads (*n* = 191)		
29	Steeman (2007)	Interview study	Elderly people with probable mild dementia and their family members (*n* = 20)		
30	Zimmerman (2015)	Interview study	Family members of decedents from 118 nursing home and residential settings (*n* = 264)		
31	McMahan (2013)	Semi-structured focus groups	Focus groups with participants from a Veterans Affairs and county hospital and the community (n = 13)		
32	Steeman (2013)	Longitudinal interview study	Elderly persons with early-stage dementia (n = 17)		
	*Mixed methods research*				
33	Silvester (2012)	Survey (1) and review of existing ACP-related documentation (2)	(1) staff of aged care facilities (*n* = 45); (2) aged care facilities (n = 12)		
34	Froggatt (2009)	Survey (1) and semi-structured interviews (2)	(1) care home managers (*n* = 213); (2) care home managers (n = 15)		
35	de Boer (2011)	Survey (1) and semi-structured interviews (2)	(1) elderly care physicians (*n* = 434); (2) physicians (n = 11) and relatives (n = 8)		
36	Van der steen (2014)	Five-round Delphi study	experts from 23 countries (*n* = 64)		
*Guidelines, reports, theses*					
37	Van Mechelen (2014)	Guideline		NA	
38	Clayton (2007)	Guideline		NA	
39	WHO (2012)	Report		NA	
40	Harle (2008)	Report		NA	
41	Titler (2008)	Guideline		NA	
42	Vellinga (2006)	Thesis		NA	
43	Church (2007)	Guideline		NA	
44	Conroy (2009)	Guideline		NA	
45	American Medical Association (1999)	Guideline		NA	
*Opinion and reflection papers*					
46	Harvey (2006)	NA		NA	
47	Lemmens (2012)	NA		NA	
48	Gillick (2012)	NA		NA	
49	Scott (2012)	NA		NA	
50	Berghmans (2001)	NA		NA	
51	Burlà (2014)	NA		NA	
52	Kim Suh (2006)	NA		NA	
53	Gillick (2004)	NA		NA	
54	Juthani-Mehta (2015)	NA		NA	
55	Mold (1991)	NA		NA	
56	Smith (2013)	hypothetical case report (n = 2)		2	
*Grey literature*					
57	Van der steen (2011)	Leaflet *(Dutch)*		NA	
58	Keirse (2009)	Leaflet *(Dutch)*		NA	

*NA* Not Applicable, *GP* General Practitioner, *IG* Intervention Group, *CG* Control Group, *AD* Alzheimer’s Disease

*Using the checklist that was developed by Cochrane Netherlands (http://netherlands.cochrane.org; only available in Dutch). It has been slightly adapted by the authors. It consists of 12 ‘yes – no - cannot answer/too little information in the paper’ questions, organised into three domains (validity, importance and applicability): 1) Was the search request adequately formulated? 2) Was the search performed adequately? 3) Was the selection procedure for the articles performed adequately? 4) Was the quality assessment performed adequately? 5) Is the description of how the data extraction was organised adequate? 6) Were the most important characteristics of the included research reported? 7) Was the meta-analysis carried out appropriately? 8) Was there statistical pooling? 9) Is the research valid? 10) Are the results adequately described? 11) Are the findings applicable in the region? 12) Is this applicable in daily practice? (translation by authors). The quality score is the number of times ‘yes’ was applied to the questions (1 ‘low quality’ - 12 ‘high quality’)

### 2) Development of recommendations

Data extraction followed a structured process in which the research questions were divided by theme and given to a pair of authors for each of the six clinical themes. Each pair reviewed a selection of the included literature and extracted data (‘key messages’) that was applicable to their clinical research question. Extracted data was stored and structured in a Microsoft Excel™ matrix. These data were then used to inform the development of a first draft of possible recommendations drawn up by two authors (GA and LVdB). Two authors (GA and JS) additionally assessed the strength of each recommendation through critical appraisal of the evidence, against the criteria of the Grading of Recommendations Assessment, Development and Evaluation (GRADE) working group [30–35]. The quality of the included literature and each recommendation can be found in Table 2 and Table 3.

**Table 3:** Recommendations

	Recommendations^a^	Quality of the recommendation, according to GRADE^b^
Domain 1	Initiation of ACP	
1	Start ACP as early as possible and integrate ACP into the daily care of people living with dementia [10, 37, 106] [11, 38–43] Specific key moments might be: - the period around the diagnosis of dementia [39, 44] - when discussing the general care plan - when changes occur in the health status, place of residence or financial situation [45]	1C
2	Be alert for triggers and opportunities to start ACP and make use of any opportunity to talk about ACP [46, 47]	1C
3	The healthcare professional should initiate ACP conversations if the person living with dementia and/or those close to them do not do this themselves [37, 44–47] [38, 45–48]	1C
4	Consider the person as an individual and consider their specific situation when starting ACP conversations [43, 49]	1C
Domain 2	Evaluation of mental capacity	
5	Always assume maximal mental capacity [50, 51]	1C
6	Consider mental capacity as a fluctuating rather than static condition [52], and stay alert for signs of loss of capacity	1C
7	Judge mental capacity task-specifically i.e. for a certain decision at a particular moment in time [11, 50, 51]	1C
8	Always stay in contact with the person him/herself and ensure their maximum participation [1]	1C
9	Assess mental capacity through formal clinical assessment: - where there is doubt or disagreement between healthcare professionals and/or family - when the decisions can have far-reaching consequences - preferably by a multidisciplinary or interdisciplinary team with experience in dementia	NA*
Domain 3	Performing ACP conversations	
10	Adjust conversation style and content to the person’s level and rhythm [59]	1C
11	Explore who the significant people in their life are and who can be involved in the ACP conversations, and explore who can become their legal representative [47, 52, 61]	1C
12	Lead the conversation but do not force it to become too formulaic or phased [59]	1C
13	Explore the person’s disease awareness and their expectations, ideas and possible misconceptions concerning the disease trajectory [5]	1C
14	Where someone lacks disease awareness or is reluctant to talk about ACP, do not insist [106, 63]	1C
15	ACP conversations can best be held on several occasions and over a longer period of time [38, 106, 45] and cover several different topics such as the broader values of the person, their experience of the present and their fears about the future and the end of life, their future care goals, specific advance decisions about the end of life, advance directives	1C
16	Try to understand the whole person living with dementia; explore their life story, important values, norms, beliefs and preferences [17, 26]	1C
17	Explore the person’s current experiences; ask what is the perception of the person living with dementia of their quality of life? What are their fears and concerns? [25, 106, 52, 65]	1C
18	Explore the person’s fears and concerns for the future and for the end of life [106]	1C
19	If possible and desirable, guide the person in formulating their care goals [49, 66]	1C
20	If possible and desirable, guide the persons with formulating specific wishes concerning specific end-of-life decisions [45]	1C
21	Explore whether the person would like to have a written advance directive or if they have made one in the past [45]	1C
Domain 4	The role and importance of those close to them	
22	Involve family or significant others as early as possible in the ACP process and inform them about the role of a surrogate decision-maker [11, 26, 41]	1C
23	Evaluate their disease awareness and inform them about the expected disease trajectory and possible end-of-life decisions [17, 25, 43, 82, 83]	1C
24	Pay attention to their perceptions during the ACP process [11, 26, 52, 65, 85]	1B
Domain 5	ACP when it is difficult or no longer possible to communicate verbally	
25	Keep connected with the person living with dementia and ensure their maximum participation [1]: respond to their emotions, attend to non-verbal communication and observe their behaviour to know more about their current quality of life, fears and desires	1C
26	Actively involve family and others close to them in the ACP process and the expression of care goals and wishes concerning end-of-life decisions [11, 26, 82]	1C
Domain 6	Documentation of wishes and preferences, including information transfer	
27	Write down in the medical/care files of the person with dementia the outcomes of the ACP process, their values, preferences and care goals, and if applicable, the advance directive and legal representative [26, 87, 88]	1B
28	Regularly re-evaluate as part of the ACP process; decisions can be revised at all times [17, 26, 47]	1C
29	Communicate the outcomes of the ACP process within the care team, i.e. values, preferences and care goals, and if applicable advance directives or legal representatives, especially in the case of transfer to another care setting.	NA*
Domain 7	End-of-life decision-making	
30	Carefully weigh the wishes (expressed and/or written down earlier) against the current best interest of the person living with dementia, in consultation with those close to them and the healthcare professionals involved [83, 89, 90]	1C
Domain 8	Preconditions for optimal implementation of ACP	
31	Provide enough training opportunities for healthcare professionals to learn how to conduct ACP conversations. Adequate support is essential in making healthcare professionals confident about engaging in ACP [11, 17, 26, 94, 114]	1C
32	Integrate ACP into the mission and policy of the organization and embed it in the organizational culture [62, 91, 95–97] [61, 96–98] [62, 96–98]	1C

*NA* Not applicable, *ACP* Advance care planning

^a^Recommendations without references were added only by the experts and end users during the consensus procedure

^b^Grading scores go from 1A to 2B, 1A representing a strong recommendation, based on a high level of evidence and 2C representing a weak recommendation and low to very low level of evidence. A grading score of 1C represents ‘strong recommendation but low to very low level of evidence’ meaning that this recommendation can be applied to patients and to care but may still change once higher-quality evidence is available. A grading score of 1B represents ‘strong recommendation and moderate level of evidence’ meaning that this recommendation has enough support for it to be applied in practice. More information on GRADE scores can be found on the website of the GRADE working group

When there was not enough evidence on a clinical research question, the author group formulated an expert opinion. Each step in the decision process was discussed and approved within the author group. The recommendations were finally re-organized into eight domains.

### 3) Validation process

Because high-quality evidence was lacking for many of the clinical research questions, we conducted additional validation of the recommendations. The results of this validation round were discussed within the author group and recommendations were revised if necessary and applicable.

1. To assess the clarity, acceptability and importance of each formulated recommendation, an online survey was set up. This survey was then e-mailed or sent with the newsletter of the Flemish Expertise Centre on Dementia Care and the Flemish Council for the Elderly to potential end users (healthcare professionals working with people living with dementia) across settings (primary care, home care, residential care and hospitals) in Flanders. The respondents were asked to score each recommendation on a scale of 1 to 7 for (i) clarity, (ii) acceptability and (iii) importance. As a result of this validation survey the authors provided more information for some of the terms used in the recommendation. For example: ‘care goals’ were defined more clearly by providing several examples such as “prolonging life, preserving function or control, optimal comfort, improving quality of life, a ‘good death’ or support from those close to them”.
2. To evaluate possible barriers to the implementation of each recommendation, we organised two meetings of established peer review groups of GPs and geriatricians on March 8 and April 28, 2016 to provide feedback. Nearly 97% of all physicians in Belgium are affiliated with peer review groups like these and are obliged to attend two out of four meetings per year for accreditation [36].
3. Finally, we provided several experts (other than the authors) with the first draft of the recommendations (informed by 1 and 2) for them to formulate comments to improve them. Experts are healthcare professionals specifically selected by the authors from different disciplines, all with an extensive knowledge of the daily practice of dementia care and ACP.

## Results

### Selection of research questions and clinical themes

The research questions and clinical themes that needed to be addressed according to the multidisciplinary author group are provided in Table 1.

### Search for evidence and expert and user validation

Figure 1 summarises the flow of selected publications through the review of all literature. A total of 67 publications constituted the evidence and validation base upon which the recommendations were developed (Table 2). In total, 51 end users, 10 experts, 12 GPs and 12 geriatricians confirmed the importance, relevance and clarity of the recommendations and helped to further define them. Characteristics of the participants in the validation process are described in Table 4.

**Table 4:** Professional background of the participants involved during the validation process

Professional background	N
Survey participants (end users)	51
Nurse	17
Dementia reference person	8
Social worker	5
Occupational therapist	4
Physician	3
Other healthcare professionals in various settings	14
Experts	10
Geriatric psychiatrist	1
Neurologist	1
Social worker	2
Nurse	2
General practitioner	1
Occupational therapist	1
Psychologist	2
Peer-review groups	2
Family physicians	12
Geriatrician	12

### Recommendations

We formulated 32 recommendations covering eight domains: 1) initiation of ACP, 2) evaluation of mental capacity, 3) holding ACP conversations, 4) the role and importance of those close to the person with dementia, 5) ACP when it is difficult or no longer possible to communicate verbally, 6) documentation of wishes and preferences, including information transfer, 7) end-of-life decision-making and 8) preconditions for optimal implementation. The main recommendations within each of the eight main domains are stated in bold and described below. The recommendations are presented in Table 3, with accompanying scores indicating their strength and supporting references.

#### Initiation of ACP

**Start ACP as early as possible and integrate ACP into the daily care of people living with dementia**, ideally before diagnosis or any cognitive decline [9, 25, 37–42]. Preferably, ACP should be performed on several occasions. These conversations can vary from short to lengthy discussions depending on how the person with dementia feels and how much time there is. They can be planned or occur spontaneously when the opportunity arises [23, 37–41, 43]. There are several key triggers for ACP conversations identified in the literature: admission to a nursing home, initiation of palliative care, deterioration of the condition or upon request. Specifically for dementia, **key moments might be the period around diagnosis** [38, 44], **while discussing the overall general care plan and/or when changes occur in health status, place of residence or financial situation** [45]. **Be alert for triggers and opportunities to start ACP and make use of any opportunity to talk about ACP** [46, 47]. Given the fluctuating cognitive capacities of people with dementia it is important to make use of spontaneous opportunities.

Because research has shown that ACP conversations are not often initiated by the person living with dementia him/herself, **healthcare professionals should initiate them** unless the person and/or those close to them do this [37, 45–47]. Although GPs play an important role, all healthcare professionals can be involved in discussing elements of ACP [46, 47] according to their own skills [37, 45–47]. It is important to have a trusting relationship with the person and those close to them, to have some knowledge of the disease trajectory [37, 48] and to communicate with the GP.

Each individual patient and situation is different. Hence, when starting ACP conversations, one needs to **consider the person as an individual and consider their specific situation** [43, 49].

#### Evaluation of mental capacity

When performing ACP with people living with dementia, their mental capacity should be considered. However, a diagnosis of dementia should not automatically be equated with loss of mental capacity. Healthcare professionals should consider the following principles:

**Always assume full mental capacity** [50, 51] and regard it **as a fluctuating, not static, condition** situated on a continuum [52]. **Stay alert for signals of loss of mental capacity. Judge mental capacity task-specifically** as the capacity for making a certain decision at a particular moment [9, 50, 51]. **Always stay in contact with the person him−/herself to ensure maximal participation** [1]. **A formal clinical assessment (including substantive clinical and neuropsychological examinations** [53]) **is only necessary in case of doubt or disagreement between healthcare professionals and/or those close to the person, or when decisions can have far-reaching consequences, and should then preferably be performed by a multidisciplinary team with expertise in dementia**. To be able to hold ACP conversations with people with dementia, a general clinical judgment of mental capacity as part of the conversation usually suffices. Available tools for making general clinical judgments of mental capacity are the MacArthur Competence Assessment Tool [54], the Vignette method [55] or the flow chart guide from Church et al. [56].

#### ACP conversations

In people with dementia, cognitive activity and abstract thinking – abilities which are needed to think about the future – can become difficult, even in mild cases [42]. Moreover, people with dementia are likely to live in the present and thinking about the future may cause fear or anxiety. This does not preclude ACP but does make ACP conversations more difficult [21, 57, 58]. To facilitate ACP conversations with people with dementia, the following recommendations apply:

When engaging in a conversation with a person who has mild/moderate dementia, **adjust the communication style and content to their own level and rhythm** [59], taking into account the principles of person-centred care [60].

**Find out who are the significant people in their life, people who may be able to be involved in the ACP conversations, and who may be able to become their surrogate decision-makers** (if not yet appointed), while explaining that these are people who can legally be appointed to act on behalf of a patient when s/he is no longer capable [47, 52, 61].

**Lead the conversation but do not make it too phased**, despite the fact that ACP is often described in such a way [59]. Because of a lack of disease awareness, decreasing decision-making ability and imaginative capacity and decreasing ability to process new information, it will often not be possible to follow a prescribed structure [25, 43, 62]. Supporting materials, if necessary and available, can be helpful (e.g. applications, books, etc.).

**Explore the person’s disease awareness and his/her expectations, ideas and possible misconceptions concerning the disease trajectory** [5]. It is important to provide a balanced view of what living with dementia may entail.

**If someone lacks disease awareness or is reluctant to talk about ACP, do not insist** [42, 63]. It is important for people to decide their own information preferences. However, even if disease awareness is lacking, it remains important to explore someone’s general values and concerns as part of the ACP process [9, 64].

**ACP conversations are best held on several occasions over a period of time** [37, 42, 45]. They can cover several different topics: the person’s more general values, their experience of the present and fears about the future and the end of life, their future care goals, their specific advance decisions about the end of life and advance directives.

**Learn to know who the person living with dementia is ‘as a whole person’**: explore their life story and most important values, norms, ideas and preferences in order to understand who the person is, what the significant events in their life have been and what gives their life meaning [15, 25, 37, 45].

**Explore people’s current experiences** in terms of quality of life, fears and concerns. ACP is not only about exploring the future, but includes a focus on the past and the present [4, 22, 63, 65]. **Explore the person’s fears and concerns for the future and for the end of life** [42, 63].

**If possible and desirable, guide the person in formulating his/her care goals** [8, 49, 66, 67] i.e. prolonging life, preserving function or control, optimal comfort, improving quality of life, a ‘good death’ or support from those close to them [67, 68]. Be aware that such care goals can change throughout the disease trajectory [58, 69, 70].

**If possible and desirable, guide the person in formulating specific wishes concerning specific end-of-life decisions** [45]. Most people with dementia do not die suddenly. Often medical decisions with regard to the provision of antibiotics, hospital admission in case of urgent health problems, resuscitation and artificial fluids are relevant [2, 71, 72]. Provide the necessary information about different possible end-of-life decisions in dementia (e.g. non-treatment decisions), and prevent misconceptions with regard to the use of resuscitation [73–76], artificial food and fluids [77] and antibiotics near the end of life [78–80].

**Explore whether the person would like to complete an advance directive or whether s/he has done so in the past** [45]. It is important to stipulate that documenting wishes formally can be relevant for people living with dementia, especially those who don’t have any close family or those who value being in control. However, professionals should be aware that in some situations, advance directives might not be specific enough to fully inform the decision-making process. Documented wishes will help guide end-of-life decision-making for physicians, other care professionals and those close to the person, and they will be most helpful if they are the result of a continuous and in-depth communication process.

These recommendations are mainly applicable to people who have mild or moderate dementia, with whom verbal communication is still possible. Part 5 focuses more on people with dementia who find it difficult or impossible to communicate verbally.

#### The role and importance of those close to the person with dementia

Because of the gradual loss of mental capacity in people living with dementia - more than in other diseases - they are often dependent on other people [81]. **Family or significant others should preferably be involved as early as possible in the ACP process and be informed about the role of a surrogate decision-maker** [9, 25, 40]. As part of the ACP process, it will be important to determine who can be involved in ACP conversations, but it can be difficult to determine when to involve them and how many people to involve. If a legal representative is appointed, they should be involved in ACP conversations [82]. If there is no legal representative, it will be useful to consider who will be the first point of contact for professionals, and how information is transferred among other family members. Every family is unique, so the involvement of family and those close to the person with dementia should be evaluated on a case-by-case basis, along with the person with dementia themselves.

**Evaluate the disease awareness of those close to the person and inform them of the expected disease trajectory and possible end-of-life decisions** [15, 23, 43, 83, 84]. The information preferences of those close to the person with dementia should also be explored. Make sure the information about the disease trajectory is correct and make sure it is balanced and qualified. In many cases the person with dementia does not experience his/her disease as something ‘negative’ in the way that the family does [82, 85].

**Pay attention to the needs of those close to the person during the ACP process** [9, 25, 63, 65, 86]. Sufficient support, education and information are important, as is addressing the concerns, experiences, expectations and fears of the family. Family can be unprepared or feel guilty [87]. Pay attention to the emotional process of family members and consider that family dynamics might change over time. It is not always easy to harmonise the views of those close to the person with dementia.

#### ACP when it is difficult or no longer possible to communicate verbally

In moderate/severe dementia, where verbal communication is difficult or no longer possible, formulating care goals or specific care preferences is difficult. **Keep a connection with the person with dementia and ensure their maximum participation** [1, 42]. **Respond to their emotions, attend to non-verbal communication and observe behaviour to understand more about their current quality of life, fears and desires** [63]. People’s emotions can give direction to the decision-making process [42]. Subsequently, **actively involve family or other close people in the ACP process and the expression of care goals and wishes concerning end-of-life decisions** [9, 25, 83] to get an understanding of the life story of the person with dementia and to interpret certain aspects of their behaviour or emotions.

#### Documentation of wishes and preferences, including information transfer

After every planned or unplanned ACP discussion, healthcare professionals should **write down the outcome in the patient’s medical/care files, e.g. the values, wishes or care goals of the person and, where relevant, details of an advance directive or legal representative** [25, 88, 89]. If the person wishes, support them in formulating specific wishes and advance decisions concerning the end of their life, explore whether they have made a formal written advance directive in the past or if they want to make one now [45] and provide information about the advantages and disadvantages of advance directives [2, 71]. It is recommended that **ACP documentation is evaluated regularly as part of the ACP process**, for example in anticipation of a ‘response shift’ [15, 25, 40, 47, 70]. **Decisions can be revised at all times**.

**The outcomes of the ACP process should be communicated within the care team**, i.e. values, preferences and care goals and any advance directives or legal representatives, particularly upon transfer to another care setting. This can be done verbally or in writing. Make sure relevant information is available to other care providers in the shared sections of the care file or is easily accessible when needed, especially upon transfer to another care setting. Information sharing should always take professional confidentiality into account [66].

#### End-of-life decision-making

Despite all good intentions, ACP cannot anticipate all possible scenarios. The disease trajectory is not always predictable and the emotional burden on those close to the person with dementia can often lead to a certain amount of confusion and lack of clarity about providing care. When end-of-life decisions need to be made, it is important to **weigh carefully the wishes expressed and/or written down earlier against the current best interest of the person living with dementia, in consultation with the person’s close circle and the healthcare professionals involved** [84, 90, 91]. End-of-life decision-making entails shared decision-making and as much consensus amongst healthcare providers and those close to the person as possible [84, 90, 91]. Materials such as the Framework for Weighing Previously Expressed Preferences v. Best Interest can support professionals and family in making these decisions, by asking questions such as: ‘is the clinical situation an emergency that allows no time for deliberation?’, ‘in view of the person’s values and goals, how likely is it that the benefits of the intervention outweigh the burdens?’, ‘to what degree does the advance directive fit the situation at hand?’, ‘how much leeway did the patient allow the surrogate in overriding the advance directive?’ or ‘how well does the surrogate represent the patient’s best interests?’ [87].

#### Preconditions for optimal implementation of ACP

The optimal implementation of ACP requires improved public understanding of end-of-life care issues [92] and patients who are more informed or educated about ACP [92]. Additionally, **the provision of sufficient training opportunities for healthcare professionals to learn how to conduct ACP conversations is important. Adequate support in practice is essential in making healthcare professionals confident about engaging in ACP** [9, 20, 25, 93, 94]. Training should at least entail the basic principles of ACP, the legal, deontological and ethical framework, the importance and effectiveness of ACP, a discussion of the professionals’ own barriers to ACP, general communication techniques and active listening skills, documentation of advance directives, communication with other professionals and how to make decisions at certain times [9, 20, 25, 93, 94]. Interactive sessions with role-plays, regular come-back sessions and a specific focus on attitudes towards talking about death and dying are also important [5, 25, 62, 95, 96]. These training programmes should be organised for GPs, nurses and social care workers, as the skills they provide often function as an important facilitator between physician and patient [92, 97].

**Integrate ACP into the mission and policy of the organization and embed ACP in the organizational culture** [62, 97–99]. ACP should be part of daily practice and this requires a supportive culture within the community or facility and an open attitude to conversations about end-of-life care and dementia among healthcare professionals. Within the facility there should be a clear statement of intent and a formal policy concerning ACP and how to embed it in routine care [1, 62, 92, 97–99].

## Discussion

There are few guidelines available for healthcare professionals concerning ACP in people living with dementia, especially those with early onset dementia. And those guidelines are often not developed using high-quality research, mainly because such research is lacking. Difficulties implementing ACP in this population and the evaluation of “active ingredients” necessary to successfully change outcomes are not fully addressed in research and high-quality evaluation research such as randomised design studies are still rare [6, 8]. By maintaining a systematic approach, we could define a unique set of recommendations to provide ACP to people living with dementia and those close to them. In doing, so we integrated the available expertise in dementia care in a wide range of settings in Flanders with the existing evidence on ACP as reported in the scientific literature.

Compared with ACP in other diseases where lack of mental capacity is a less pronounced problem, performing ACP in dementia entails several significant and specific attention points. The most important concerns the involvement of those close to the person with dementia. Family members, next-of-kin and other significant people are an important point of contact in communication and decision-making in end-of-life care for people with dementia, as their mental capacity gradually declines and verbal communication becomes more difficult or even impossible [1]. Involvement of these people from the initial stages of the condition is of the utmost importance in providing end-of-life care that corresponds to the wishes and preferences of the person with dementia.

A second element very specific to ACP in people with dementia is the trajectory of the decrease in mental capacity [81]. The clinical question of the evaluation of mental capacity as part of the ACP process was heavily debated by the author group and within the expert panel and proved to be something which is difficult to do in practice. Because of these discussions, we concluded that mental capacity should be considered as a continuum that fluctuates over time and is task-specific. In addition, we recommend that formal, in-depth, multidisciplinary assessments of capacity should not always be performed before or during ACP conversations. However, it is important that care professionals hold ACP conversations at different points over a period of time, making use of spontaneous remarks on ACP-related issues by patients or those close to them, as well as having planned conversations. Such conversations will not always follow a predefined or structured format and will vary in content, length and depth depending on the physical, cognitive and psychological state of the person. In some cases, e.g. when high-stake decisions need to be made, formal multidisciplinary assessment and referral will be necessary. However, further research is needed to substantiate this recommendation.

ACP is an important part of care, especially in older people and those living with dementia. Older people themselves indicate that they find ACP valuable [17, 63] and there is an important body of literature suggesting that it has a positive impact on outcomes, ranging from family satisfaction with care to concordance between end-of-life care and patient wishes, especially for older people and those living in nursing homes [100, 101]. There is additional evidence, albeit of variable quality, which shows that ACP has the potential to reduce inappropriate hospital admissions and healthcare costs in nursing homes where end-of-life care spending is already high [39, 102]. Further high-quality research, however, would strengthen the arguments for ACP becoming part of routine dementia care and provide information on how it can be carried out effectively and sustainably [103]. More specifically, we found insufficient research to support recommendations on issues such as a uniform definition of lack of mental capacity, contraindications for initiating ACP and what to do if a person living with dementia does not want to involve those close to them.

To the best of our knowledge, this is the first practical guideline developed to improve the performance of healthcare professionals in providing ACP to people living with dementia across settings. Until now, existing guidelines from guideline development groups (GDGs) such as the National Institute for Health and Clinical Excellence have only highlighted a few recommendations concerning advance care planning, and these are limited to ‘discuss the use of advance directives and identify surrogates’ and ‘discuss cardiopulmonary resuscitation in advance and inform patients about poor outcomes in advanced dementia’ [26, 104]. Nonetheless, evidence shows that ACP may be more effective in meeting a patient’s preferences when it entails more than just written documents and a conversation [39, 101]. Local initiatives have tried to provide guidance in ACP specifically for people living with dementia, but standardization and consistency are lacking [105]. By consulting both experts and end users with different professional backgrounds, we have been able to include broad multidisciplinary support at a regional level for these recommendations. In addition, rather than just requiring the experts to agree or disagree with predefined statements, they were actively involved in specifying the statements so that greater consensus could be achieved. We consider this an important feature of our work.

However, readers must be aware of several limitations of this study. The first limitation is the rather small number of experts (*n* = 10) and end users (*n* = 51) who replied to the survey, and the limited number of peer-review groups (*n* = 2). In addition*,* the main source from which the recommendations are derived is low-quality systematic reviews, studies in which the quality of evidence was not assessed formally, and from the opinions of professionals or experts. We also wish to make clear that professionals must be aware of the policies and legislation that govern the jurisdiction in which they work and that they must abide by existing policies and legislation when applying the recommendations. Additionally, healthcare professionals should of course apply these recommendations in their workplaces to serve as a general guide, but follow them subject to their own judgment and each individual case. The recommendations serve neither as an action programme nor as a strict guideline but provide a list of attention points for healthcare professionals involved in dementia care. We recommend that they should additionally be trained to perform ACP, because merely providing and disseminating a guideline like this will not be enough to improve their practice [106]. The results of this study can serve as a tool to educate healthcare professionals.

The final guideline requires further testing in clinical practice. However, initial feedback from experienced healthcare professionals and other experts has indicated that it can be helpful in terms of initiation, organisation and implementation of ACP and when holding discussions on end-of-life care. Such guidelines are shown to play an important role in enabling good care up to the end of life which is provided according to high ethical and quality standards [1].

## Conclusion

Little high-quality evidence is available on ACP in dementia care. By combining the available evidence with expert and user opinions, we have defined a unique set of recommendations for ACP in people living with dementia. These recommendations will be used for the development of a Flemish guideline for ACP in people living with dementia and can serve as a valuable tool to educate healthcare professionals on how to perform ACP across settings.

## Abbreviations

ACP: Advance care planning
CAP: Coordinating and advising physician
GP: General practitioner
